# Cerebrospinal Fluid and Microdialysis Cytokines in Aneurysmal Subarachnoid Hemorrhage: A Scoping Systematic Review

**DOI:** 10.3389/fneur.2017.00379

**Published:** 2017-08-08

**Authors:** Frederick A. Zeiler, Eric Peter Thelin, Marek Czosnyka, Peter J. Hutchinson, David K. Menon, Adel Helmy

**Affiliations:** ^1^Rady Faculty of Health Sciences, Department of Surgery, University of Manitoba, Winnipeg, MB, Canada; ^2^Clinician Investigator Program, Rady Faculty of Health Sciences, University of Manitoba, Winnipeg, MB, Canada; ^3^Department of Anesthesia, Addenbrooke’s Hospital, University of Cambridge, Cambridge, United Kingdom; ^4^Division of Neurosurgery, Department of Clinical Neurosciences, University of Cambridge, Cambridge Biomedical Campus, Cambridge, United Kingdom; ^5^Department of Clinical Neuroscience, Karolinska Institute, Stockholm, Sweden

**Keywords:** subarachnoid hemorrhage, systematic review, cytokines, cerebrospinal fluid, micordialysis

## Abstract

**Objective:**

To perform two scoping systematic reviews of the literature on cytokine measurement in cerebral microdialysis (CMD) and cerebrospinal fluid (CSF) in aneurysmal subarachnoid hemorrhage (SAH) patients, aiming to summarize the evidence relating cytokine levels to pathophysiology, disease progression, and outcome.

**Methods:**

Two separate systematic reviews were conducted: one for CMD cytokines and the second for CSF cytokines.

**Data sources:**

Articles from MEDLINE, BIOSIS, EMBASE, Global Health, Scopus, Cochrane Library (inception to October 2016), reference lists of relevant articles, and gray literature were searched.

**Study selection:**

Two reviewers independently identified all manuscripts utilizing predefined inclusion/exclusion criteria. A two-tier filter of references was conducted.

**Data extraction:**

Patient demographic and study data were extracted to tables.

**Results:**

There were 9 studies identified describing the analysis of cytokines *via* CMD in 246 aneurysmal SAH patients. Similarly, 20 studies were identified describing the analysis of CSF cytokines in 630 patients. The two scoping systematic reviews demonstrated the following: (1) limited literature available on CMD cytokine measurement in aneurysmal SAH with some preliminary data supporting feasibility of measurement and potential association between interleukin (IL)-6 and patient outcome. (2) Various CSF measured cytokines may be associated with patient outcome at 3–6 months, including IL-1ra, IL-6, IL-8, and tumor necrosis factor-alpha. (3) There is a small literature body supporting an association between acute/subacute CSF transforming growth factor levels and the development of chronic hydrocephalus at 2–3 months.

**Conclusion:**

The evaluation of CMD and CSF cytokines is an emerging area of the literature in aneurysmal SAH. Further large prospective multicenter studies on cytokines in CMD and CSF need to be conducted.

## Introduction

Inflammation in the setting of aneurysmal subarachnoid hemorrhage (SAH) is believed to be a potential driver of many secondary insults in this often critically ill population (1–4). As with animal models of stroke, inflammatory mediators have been associated with loss of salvageable ischemic penumbra, infarct propagation, and cerebral edema (5–8). It has been proposed that various inflammatory cytokines are associated with secondary injury and pathological processes post-aneurysmal SAH (1, 2).

Within the aneurysmal SAH population, numerous studies have been published associating serum inflammatory markers with patient outcome and the risk of cerebral vasospasm/delayed ischemic neurological deficits (DINDs) (1, 3, 4). Furthermore, analysis of cerebrospinal fluid (CSF) cytokines has demonstrated an association between interleukin (IL)-6 and tumor necrosis factor-alpha (TNF-a) with the risk of cerebral vasospasm and DIND through an extensive systematic review/meta-analysis of the available literature (9). Of note, this systematic review focused only on the relationship IL-6 and TNF-a with vasospasm, without any comment or consideration of other important primary outcomes such as patient morbidity/mortality.

Aside from the above-mentioned associations, the remaining literature on inflammatory cytokines in aneurysmal SAH is scattered and scarce. In particular, the literature on cytokines in cerebral microdialysis (CMD) samples from aneurysmal SAH patients is very limited (10–18). In addition, apart from the systematic review on the association between CSF IL-6 and TNF-a with vasospasm/DIND (9), there is limited literature on the association between CSF cytokines and other relevant endpoints (10, 19–37) such as patient functional outcome, neurophysiologic outcome, chronic hydrocephalus/ventriculoperitoneal shunt (VPS) dependency, and tissue fate.

The goal of this project was to produce a scoping systematic review of the literature on both CMD and CSF cytokines in aneurysmal SAH. There were two main focuses for this article: (1) to provide a comprehensive scoping systematic review of the literature on CMD cytokines in aneurysmal SAH; (2) produce a scoping systematic review evaluating the association between CSF cytokines and the following outcomes (excluding vasospasm/DIND): patient functional outcome, neurophysiologic outcome, chronic hydrocephalus/VSP dependency, and tissue outcome.

## Methods

Two separate scoping systematic reviews were conducted, using the methodology outlined in the Cochrane Handbook for Systematic Reviewers (38). Data were reported following the Preferred Reporting Items for Systematic Reviews and Meta-Analyses (39). The review questions and search strategy were decided upon by the primary author (Frederick A. Zeiler) and supervisors (Adel Helmy and David K. Menon).

This article was conducted in concert with a similar review on cytokines in CMD and CSF for severe traumatic brain injury (TBI) patients, which is currently unpublished and under review (40).

### Search Question and Population of Interest

Given that two separate systematic reviews were conducted, one for CMD cytokines and the other for CSF-based cytokines, two distinct questions were posed. The lack of literature identified through a preliminary search of PubMed led us to conduct a scoping review for the CMD cytokine search, with the attempt to identify all studies in this area to date. The larger literature base for CSF cytokines in aneurysmal SAH led us to narrow our question for this scoping review, focusing on relevant outcomes (see below). The questions posed for this scoping systematic review were as follows:

1. What literature has been published on CMD of cytokines in aneurysmal SAH?
2. Is there literature to suggest an association between CSF-based cytokine measures in aneurysmal SAH and patient outcome, chronic hydrocephalus/shunt dependency, neurophysiologic outcome, or tissue outcome?

For the CMD cytokine scoping review, all articles describing microdialysis-based cytokine measures in humans with aneurysmal SAH were included to provide a comprehensive overview.

For the CSF cytokine review, the primary outcome measures of interest were documented association between CSF measured cytokines and patient outcome, chronic hydrocephalus/shunt dependency, neurophysiologic outcome (as measured *via* intensive care unit (ICU)-based monitoring, intracranial pressure/cerebral perfusion pressure, brain tissue oxygen monitoring (PbtO_2_), thermal diffusion assessment of cerebral blood flow (CBF), transcranial Doppler (TCD) measure of cerebral blood flow velocity, any neuroimaging-based assessment of CBF/perfusion, and electrophysiology), and tissue outcome [as assessed on follow-up neuroimaging by either computed tomography (CT) or magnetic resonance imaging]. Any outcome score or mention of morbidity/mortality within the studies was deemed acceptable for documentation of patient outcome. Secondary outcome measures were complications associated with CSF monitoring of cytokines. Of note, cerebral vasospasm and DIND were specifically excluded as a primary outcome for the CSF cytokine review given a recently conducted systematic review published on this exact relationship (9).

Acceptable cytokines in CMD or CSF included IL-1a, IL-1b, IL-1ra, IL-2, sIL-2ra, IL-3, IL-4, IL-5, IL-6, IL-7, IL-8, IL-9, IL-10, IL-11, IL-12, IL-12p70, IL-13, IL-14, IL-15, IL-16, IL-17, inducible protein-10, eotaxin, TNF, interferon gamma, monocyte chemoattractant proteins (MCP), macrophage inflammatory proteins (MIPs), transforming growth factor (TGF), nerve growth factor, brain-derived neurotrophic factor, glial-derived neurotrophic factor, soluble tumor necrosis factor receptor (sTNFR), granulocyte macrophage colony-stimulating factor (GM-CSF), sFAS, soluble vascular cell adhesion molecule-1, soluble intracellular adhesion molecule-1, platelet-derived growth factor, RANTES, macrophage-derived chemokine (MDC), Flt3, fractalkine, and fibroblast growth factor receptor.

### Inclusion/Exclusion Criteria

#### CMD Cytokine Review

Inclusion criteria: all studies including human subjects with aneurysmal SAH, any study size, any age category, CMD analysis for cytokines, and mention of any outcome (patient based or otherwise; excluding vasospasm/DIND). Exclusion criteria: non-English studies, only non-cytokine/chemokine inflammasome proteins measured, animal studies, non-aneurysmal SAH, and unconfirmed/angio-negative SAH.

#### CSF Cytokine Review

Inclusion criteria were as follows: all studies including human subjects with aneurysmal SAH, studies with 10 or more patients, any age category, CSF analysis for cytokines, and documentation either: patient functional outcome, neurophysiologic outcome, or tissue outcome in relation to CSF cytokine measures. Exclusion criteria were as follows: non-English studies, only non-cytokine/chemokine inflammasome proteins measured, animal studies, non-aneurysmal SAH, unconfirmed/angio-negative SAH, and studies of less than 10 patients. Also excluded were studies focused only on the outcome of cerebral vasospasm/DIND (as this has been explored in a recent systematic review and meta-analysis).

### Search Strategies

MEDLINE, BIOSIS, EMBASE, Global Health, SCOPUS, and Cochrane Library from inception to October 2016 were searched using individualized search strategies. The search strategy for the CMD scoping systematic review using MEDLINE can be seen in Appendix A in Supplementary Material, with a similar search strategy utilized for the other databases. Further, the search strategy for the CSF scoping systematic review using MEDLINE can be seen in Appendix B in Supplementary Material, with similar strategies employed for the other databases.

In addition, we surveyed relevant meeting proceedings for the last 5 years looking for ongoing and unpublished work based on the cytokine analysis *via* CMD or CSF in aneurysmal SAH patients. We elected to include published meeting proceedings to provide as comprehensive of a scoping systematic review as possible. It is acknowledged that the quality of evidence derived from such pseudopeer-reviewed meeting publications is limited. However, given that cytokine research in SAH is relatively “new” and our goal was to produce a systematically conducted scoping review on the topic, we elected to include them to be comprehensive. The meeting proceedings of the following professional societies were searched: Canadian Neurological Sciences Federation, American Association of Neurological Surgeons, Congress of Neurological Surgeons, European Neurosurgical Society, World Federation of Neurological Surgeons (WFNS), American Neurology Association, American Academy of Neurology, European Federation of Neurological Science, World Congress of Neurology, Society of Critical Care Medicine, Neurocritical Care Society, European Society for Intensive Care Medicine, World Federation of Societies of Intensive and Critical Care Medicine, American Society for Anesthesiologists, World Federation of Societies of Anesthesiologist, Australian Society of Anesthesiologists, International Anesthesia Research Society, Society of Neurosurgical Anesthesiology and Critical Care, Society for Neuroscience in Anesthesiology and Critical Care, Japanese Society of Neuroanesthesia and Critical Care, and the College of Intensive Care Medicine Annual Scientific Meeting (Australia), World Stroke Organization, UK Stroke Forum, International Stroke Conference, European Stroke Society, Canadian Stroke Congress, SMART Stroke Group, and the Australian Stroke Society.

Finally, reference lists of any review articles on CSF or CMD cytokines were reviewed for any missed relevant studies.

### Study Selection

Utilizing two reviewers, a two-step review of all articles returned by our search strategies was performed. First, the reviewers independently (Frederick A. Zeiler and Eric Peter Thelin) screened titles and abstracts of the returned articles to decide if they met the inclusion criteria. Second, full text of the chosen articles was then assessed to confirm if it met the inclusion criteria and that the primary outcome of interest was reported in the studies (Frederick A. Zeiler and Eric Peter Thelin). Any discrepancies between the two reviewers were resolved by a third reviewer if needed (Adel Helmy or David K. Menon).

### Data Collection

Data were extracted from the selected articles and stored in an electronic database. Data fields included type of study, article location, number of patients, patient demographics, aneurysm characteristics/treatment, Hunt and Hess (H + H) clinical grade (41), World Federation of Neurological Surgeons (WFNS) clinical grade (42), Fisher CT grade (43), ICU therapies applied, CMD/CSF substrate measured, CMD/CSF measurement details (probe tissue location and sampling frequency), outcome measure described (patient, neurophysiologic, and tissue), association between CMD/CSF cytokine measure to outcome, and complications. Complications of interest for the CSF studies were any related to ventriculostomy: misplacement, tract hemorrhage, infection, and extra-axial hemorrhage/collection formation. All data for both the CSF and CMD cytokine studies can be found in Tables 1–4.

**Table 1:** CMD cytokine study characteristics and patient demographics.

Reference	Number of patients	Study type	Article location	Mean age (years)	Patient characteristics	Primary and secondary goal of study
Graetz et al. (10)	24	Prospective observational	Manuscript	50 years (range: 43.5–62 years)	aSAH	*Primary*: to evaluate the pattern of IL-6 expression in CMD, CSF, and serum
					Admission WFNS: I–III in 14 IV–V in 10	
					Mean Fisher CT Score: 4 (range: 3–4)	*Secondary*: compare IL-6 expression to ICP, CMD-defined ischemia (LPR > 30, glycerol > 80 μmol/L), and outcome
					Aneurysm location ICA/MCA = 5/13 ACA/PComm = 3/3	

Hanafy et al. (11)*	14	Prospective observational	Manuscript	48 years (range: 34–59 years)	aSAH	*Primary*: to measure CMD TNF-a post-aSAH
					Admission WFNS: IV in 1 V in 13	*Secondary*: to determine if clinical characteristics predict CMD TNF-a levels
					Aneurysm locations:	
					ICA (4); MCA (3); ACA (6); VA (1)	

Hanafy et al. (12)*	10	Retrospective case series	Manuscript	45.5 years (range: 27–65 years)	aSAH	*Primary*: to determine the correlation between CMD TNF-a and radiographic vasospasm as per CTA/DSA
					Admission H + H: 2 in 1 3 in 1 4 in 3 5 in 5	
					Fisher CT:	*Secondary*: none mentioned
					Median = 3 (range: 2–4)	
					Aneurysm locations: AComm (3); ICA (3); MCA (2); PCA (1); VA (1)	

Helbok et al. (13)	26	Prospective observational	Manuscript	55 years (range: 47–67 years)	aSAH	*Primary*: to measure CMD IL-6 and CMD MMP-9 and determine the relationship to outcome
					Admission H + H: 2 in 2 3 in 6 4 in 2 5 in 16	*Secondary*: to determine the temporal course of IL-6 post-aSAH
					Aneurysm location: unclear locations	

Mellergård et al. (14)	21 with aSAH (38 total in study with mixed pathology)	Prospective observational	Manuscript	Unknown	aSAH	*Primary*: to evaluate CMD cytokine profiles immediately after insertion of the CMD catheter
					No specifics on clinical status or aneurysms	*Secondary*: none mentioned

Mellergård et al. (15)*	88 with aSAH (Total 145 patients with mixed pathology)	Retrospective case series	Manuscript	Unknown	aSAH	*Primary*: to determine the CMD cytokine responds to aSAH
					No specifics on clinical status or aneurysms	*Secondary*: none mentioned

Mellergård et al. (16)*	88 with aSAH (total 145 patients with mixed pathology)	Retrospective case series	Manuscript	Unknown	aSAH	*Primary*: to determine the CMD cytokine response to aSAH
					No specifics on clinical status or aneurysms	*Secondary*: none mentioned

Sarrafzadeh et al. (17)	38	Prospective observational	Manuscript	53.1 years (range: unknown)	aSAH—29% with acute focal deficits on admission	*Primary*: to measure CMD, CSF and serum IL-6 post-aSAH
					Admission WFNS scores: I in 12 II in 7 III in 3 IV in 7 V in 9	
					Mean Fisher CT score = 4	*Secondary*: correlate to clinical course
					Aneurysm locations: no specific given	

Schiefecker et al. (18)	25	Prospective observational	Manuscript	Unknown	aSAH—poor grade	*Primary*: to evaluate CMD IL-6 levels post-aSAH and determine the association DIND and outcome at 3 months
						*Secondary*: determine probe relationship to IL-6 expression

*Studies from the same Authors and Center – there may be duplicated patient information.

*aSAH, aneurysmal subarachnoid hemorrhage; H + H, Hunt and Hess; WFNS, World Federation of Neurological Surgeons; CT, computed tomography; AComm, anterior communicating artery; PComm, posterior communicating artery; MCA, middle cerebral artery; ACA, anterior cerebral artery; ICA, internal cerebral artery; VA, vertebral artery; VBA, vertebrobasilar; PICA, posterior inferior cerebellar artery; CMD, cerebral microdialysis; CSF, cerebrospinal fluid; ICP, intracranial pressure; LPR, lactate:pyruvate ratio; IL, interleukin; MABP, mean arterial blood pressure; DC, decompressive craniectomy; DIND, delayed ischemic neurological deficit; PCA, principle component analysis*.

**Table 2:** CSF cytokine study characteristics and patient demographics.

Reference	Number of patients	Study type	Article location	Mean age (years)	Patient characteristics	Primary and secondary goal of study
Chou et al. (19)	29	Prospective observational	Meeting abstract	Unknown	aSAH	*Primary*: to determine the association between CSF cytokines with vasospasm and patient outcome
					Unknown admission clinical/radiologic grades	*Secondary*: none mentioned
					No data on aneurysm characteristics	

Graetz et al. (10)	24	Prospective observational	Manuscript	50 years (range: 43.5–62 years)	aSAH	*Primary*: to evaluate the pattern of IL-6 expression in CMD, CSF, and serum
					Admission WFNS: I–III in 14 IV–V in 10	
					Mean Fisher Score: 4 (range: 3–4)	*Secondary*: compare IL-6 expression to ICP, CMD-defined ischemia (LPR > 30, glycerol > 80 μmol/L), and outcome
					Aneurysm Location: ICA/MCA = 5/13 ACA/PComm = 3/3	

Gruber et al. (20)	44	Prospective observational	Manuscript	51.3 years (range: 24–80 years)	aSAH	*Primary*: to measure CSF and serum cytokines. Determine any association to outcome.
					Admission H + H: I = 2 II = 4 III = 15 IV = 19 V = 4	*Secondary*: none mentioned
					Aneurysm location: Ant Circ = 30 Post Circ = 14	

Höllig et al. (21)	46 (total 81; only 46 with CSF sampling)	Prospective observational	Manuscript	53.8 years (range: 29–87 years)	aSAH	*Primary*: to determine the relation of serum and CSF cytokines with discharge outcome.
					Admission WFNS:	*Secondary*: to determine the relation of serum and CSF cytokines with 6-month outcome
					Mean = 2.96	
					Admission Fisher Score:	
					Mean = 3.31	
					Aneurysm location: AComm = 26 MCA = 17 ICA = 19 BA = 7 “other” = 12	

Mathiesen et al. (22)	22	Prospective observational	Manuscript	51.3 years (range: 32–77 years)	aSAH	*Primary*: to measure CSF cytokines in SAH patients and correlate to outcome and vasospasm.
					Admission H + H: I = 0 II = 14 III = 3 IV = 5	*Secondary*: none mentioned
					Fisher CT score:	
					Not specified	
					Aneurysm location: AComm = 9 MCA = 6 PComm = 3 ICA = 2 VA/VAB = 2	

Nakahara et al. (23)	39	Prospective observational	Manuscript	62.9 years (range: 52–71)	aSAH	*Primary*: to determine the correlation between HMGB-1 and other cytokines with patient outcome at 3 months
					Admission H + H:	*Secondary*: none mentioned
					Range = 2–4	
					Fisher CT score:	
					Range = 3–4	
					Aneurysm location:	
					AComm = 12MCA = 12ICA/PComm = 11ACA = 2BA = 2	

Niwa et al. (24)	10	Prospective observational	Manuscript	57 years (range: 41–75 years)	aSAH	*Primary*: to measure various CSF cytokines post-SAH. Determine association to patient outcome
					Admission H + H: I = 0 II = 6 III = 2 IV = 2 V = 0	*Secondary*: none mentioned
					Fisher CT score: I = 0 II = 0 III = 7 IV = 3	
					Aneurysm location:	
					AComm = 6MCA = 2ICA/PComm = 2	

Provencio et al. (25)	14	Prospective observational	Meeting abstract	Unknown	aSAH	*Primary*: to determine the relationship between serum and CSF cytokines with patient outcome
					Unknown admission clinical/radiologic grades	*Secondary*: none mentioned
					No data on aneurysm characteristics	

Sokół et al. (26)	10	Prospective observational	Manuscript	61.1 years (range: unknown)	aSAH	*Primary*: to determine the association between CSF HMGB-1 and patient outcome
					Admission H + H:	*Secondary*: none mentioned
					Mean = 4 (range: 4–4)	
					Admission Fisher CT score:	
					Mean = 4 (range: 2–4)	
					Aneurysm location: AComm = 4 ACA = 2 BA = 2 PCA = 1 PICA = 1	

Wada et al. (27)	45	Prospective observational	Meeting abstract	Unknown	aSAH	*Primary*: to determine the association between serum/CSF G-CSF and both vasospasm and patient outcome
					Unknown admission clinical/radiologic grades	*Secondary*: none mentioned
					No data on aneurysm characteristics	
**SHUNT DEPENDENCY STUDIES**						
Douglas et al. (28)	20	Prospective observational	Manuscript	47 years (range: 23–64 years)	aSAH	*Primary*: to determine the association between CSF TGF-b1 and TGF-b2 with shunt dependency post-aSAH
					Admission WFNS: I = 5 II = 4 III = 1 IV = 2 V = 6 ND = 2	*Secondary*: to compare CSF cytokines in aSAH and non-hemorrhage hydrocephalus patients
					Admission Fisher CT score: I = 1 II = 2 III = 3 IV = 11 ND = 3	
					Aneurysm location: ACA = 3 ICA = 2 PCA = 1 PICA = 3 BA = 1 Traumatic = 1 ND = 8	

Kitazawa and Tada (29)	24	Prospective observational	Manuscript	61.2 years (range: 39–78 years)	aSAH	*Primary*: to determine the association between CSF TGF-b1 levels and the development of CT-defined hydrocephalus and VPS dependency
					unknown admission clinical grades	
					Admission Fisher CT grade: II = 5 III = 14 IV = 3	
						*Secondary*: none mentioned
					Aneurysm locations: unknown	

Takizawa et al. (30)	36	Prospective observational	Manuscript	60.3 years (range: 39–81 years)	aSAH	*Primary*: to determine the association between CSF measured cytokines and the development of hydrocephalus
					Unknown admission clinical/radiologic grades	*Secondary*: compare levels to non-hemorrhagic controls (*n* = 11)
					No data on aneurysm characteristics	

Wostrack et al. (31)	69	Prospective observational	Manuscript	57 years (range: 21–80 years)	aSAH	*Primary*: to determine the association between CSF cytokines and shunt dependency
					Admission H + H: I = 3 II = 17 III = 25 IV = 12 V = 12	
					Unclear Fisher grades	*Secondary*: none mentioned
					Aneurysm locations: Anterior = 23 Middle = 16 ICA = 16 Posterior = 10	
**NIL ASSOCIATION STUDIES**						
Gaetani et al. (32)	31	Prospective observational	Manuscript	52.6 years (range: unknown)	aSAH	*Primary*: to determine the association between various CSF cytokines and the development of vasospasm
					Admission WFNS: 1–2 = 24 3–4 = 7	
					Unclear Fisher grade and aneurysm Locations	

Kaestner and Dimitriou (33)	27 (42 total; but non-aneurysmal IVH in 15 patients)	Prospective observational	Manuscript	52.2 years (range: unknown)	aSAH	*Primary*: to determine the association between CSF TGF-b1 and hydrocephalus
					Unknown admission clinical/radiologic grades	*Secondary*: to compare CSF TGF-b1 with non-hemorrhagic communicating hydrocephalus (*n* = 7)
					Unknown aneurysm locations	

Kim et al. (34)	51 (77 total; only 51 with CSF samples)	Prospective observational	Manuscript	Unclear mean and range	aSAH	*Primary*: to determine the association between serum and CSF MIP-1 with patient outcome
					Unknown admission clinical/radiologic grades	*Secondary*: to determine the association between serum and CSF MIP-1 with vasospasm
					No data on aneurysm characteristics	

Kwon and Jeon (35)	12 (19 patients total; 12 with CSF)	Prospective observational	Manuscript	46.5 years (range: 29–65 years)	aSAH	*Primary*: to determine the relationship between serum/CSF cytokines and vasospasm. Also determine the link to patient outcome
					Admission H + H: I = 2 II = 6 III = 8 IV = 3	*Secondary*: none mentioned
					Admission Fisher score: I = 3 II = 5 III = 9 IV = 2	
					Aneurysm characteristics: ACom = 8 MCA = 3 PCom = 5 ICA = 1 ACA = 1 VA = 1	

Shoch et al. (36)	64	Prospective observational	Manuscript	55 years (range: 29–77 years)	aSAH	*Primary*: to determine the association between CSF IL-6 and vasospasm
					Admission WFNS: I = 9 II = 12 III = 4 IV = 25 V = 14	*Secondary*: none mentioned
					Admission Fisher CT Score: I = 0 II = 4 III = 32 IV = 28	
					Aneurysm Location:	
					ACA = 23MCA = 12AComm = 15VBA = 14	

Singh et al. (37)	13	Prospective RCT	Manuscript	54 years (range: 40–69 years)	aSAH	*Primary*: to evaluate the use of IV IL-1ra in aSAH and evaluated CSF cytokine response
					Admission WFNS: I = 1 II = 5 III = 0 IV = 3 V = 4	*Secondary*: to evaluate patient outcome
					Admission Fisher CT score: III = 3 IV = 10	
					Aneurysm location: Anterior = 9 Posterior = 4	

*aSAH, aneurysmal subarachnoid hemorrhage; H + H, Hunt and Hess; WFNS, World Federation of Neurological Surgeons; RCT, randomized control trial; CT, computed tomography; VPS, ventriculoperitoneal shunt; IVH, intraventricular hemorrhage; AComm, anterior communicating artery; PComm, posterior communicating artery; MCA, middle cerebral artery; ACA, anterior cerebral artery; ICA, internal cerebral artery; VA, vertebral artery; VBA, vertebrobasilar; PICA, posterior inferior cerebellar artery; CMD, cerebral microdialysis; CSF, cerebrospinal fluid; ICP, intracranial pressure; LPR, lactate:pyruvate ratio; IL, interleukin; TGF, transforming growth factor; G-CSF, granulocyte colony-stimulating factor; HMGB, high-mobility group box; MABP, mean arterial blood pressure; DC, decompressive craniectomy; PCA, principle component analysis*.

**Table 3:** CMD cytokine measures and outcomes.

Reference	Catheter location and measured CMD cytokines	Interventional therapies applied during measurement	Primary outcome	Secondary outcome	Complications to CMD	Conclusions
Graetz et al. (10)	Inserted into territory of aneurysm (whether healthy or injured)	Protocolized therapy for monitoring and Tx of ICP; 3 patients underwent DC	IL-6 in CSF and CMD were typically higher than in serum	*ICP*: CMD and CSF IL-6 levels were higher in the high ICP patients, with significant for CMD samples (*p* = 0.029)	Not specified	IL-6 levels in CSF, CMD, and serum are elevated after aSAH CMD IL-6 levels are higher in those with ICP issues No correlation between CMD IL-6 and ischemia Potential weak association between CMD IL-6 levels and outcome at 3 and 6 months
	*IL-6*			CMD IL-6 levels increased after day 4 in the high ICP group		
	Unclear pooled analysis over a 10 days period			*Ischemia (as per LPR > 30 and glycerol > 80 μmol/L)*: no correlation between CMD, CSF, or serum IL-6 and ischemia		
	Ringer’s perfusate utilized	20 patients clipped		*Outcome (dichotomized GOS at 3 and 6 months)*: high CMD IL-6 levels were associated with poor outcome (*p* = 0.06)		

Hanafy et al. (11)*	Unclear tissue location	Unclear DIND monitoring; various ICP/CPP directed therapies	TNF-a as measured *via* CMD is feasible and elevated post-SAH	Only the existence of IVH and aneurysm size >6 mm was correlated to TNF-a levels in CMD	Not specified	TNF-a is elevated in CMD post-aSAH
	*TNF-a*					
	q6 hour sampling for unclear duration	Unclear Surgical Tx				IVH and large aneurysm size is associated with elevated CMD TNF-a levels
	Isotonic crystalloid perfusate					

Hanafy et al. (12)*	Unclear tissue location	Not specified; unclear surgical Tx	Increase CMD TNF-a between days 4 and 6 post-hemorrhage was associated with a worse radiographic vasospasm index (*p* < 0.01)	N/A	Not specified	Elevated CMD TNF-a levels may correlation with radiographic vasospasm
	*TNF-a*					
	q6 hour sampling for unclear duration		No comments were made on the relationship to DIND secondary to cerebral vasospasm			
	Isotonic crystalloid perfusate					

Helbok et al. (13)	“Right frontal” in mixed tissue states	Protocolized investigations for vasospasm, otherwise unclear ICU treatments	*3-month mRS*	No correlation between IL-6 and MMP-9 *via* CMD	Not specified	CMD IL-6 may be associated with outcome at 3 months
	*IL-6*					
	Unclear sampling interval	18 patients clipped; some had DC	CMD IL-6 and LPR were higher in those patients with worse mRS at 3 months (*p* = 0.01)	IL-6 was highest initially after bleed and in cases where rebleed occurred		
	Isotonic crystalloid perfusate					

Mellergård et al. (14)	Mixed locations; some patients with 2 catheters (unclear which patients)	Not specified	IL-1b peaked in the first 12 h period	N/A	Not specified	CMD catheter insertion leads to IL-1b/IL-6/IL-8/MIP-1b within the first 6–12 h, which then decrease during the subsequent time afterward
	*IL-1b, IL-6, IL-8, FGF-2, MIP-1*β, *RANTES, VEGF, IL-10*		IL-6 peaked after 12 h post-insertion			
	q6 hour pooled samples for 36 h		IL-8 peaked within the first 6 h post-insertion			
	Ringer-Dextran 60 perfusate		MIP-1b peaked within the first 6 h post-insertion			
			FGF-2 peaked within the first 6 h post-insertion			
			IL-10, VEGF, and RANTES did not show a temporal profile			

Mellergård et al. (15)*	Some with paired catheters (1 peri-lesonal; 1 healthy tissue)—used the catheter with highest glycerol levels for measuring cytokines	Not Specified	IL-1b increased during the first 48 h and then decreased	N/A	Not specified	IL-1b and IL-6 display a peak elevation during the first 48 h post-aSAH
	*IL-1b, IL-6, IL-10*		IL-6 increased over the first 48 h and then decreased			
	q6 hour pooled analysis for 7 days	65 patients clipped	IL-10 remained elevated throughout the measurement period			IL-10 remains elevated through the first 7 days post-aSAH
	Ringer-Dextran 60 perfusate		No comments on cytokine profiles in clipping vs. coiling patients were made			

Mellergård et al. (16)*	Paired catheters (1 perilesonal; 1 healthy tissue)—used the catheter with highest glycerol levels for measuring cytokines	Not specified	FGF-2 levels peaked at day 3 post-TBI	N/A	Not specified	FGF-2/VEGF levels peaked on days 3 and 2 post-aSAH
	*FGF-2, VEGF*					
	q6 hour pooled analysis for 7 days	65 patients clipped	VEGF levels peaked on day 2 post-aSAH and were higher in those whom underwent surgical clipping			Surgical clipping changes the inflammatory mediator expression in CMD
	Ringer-Dextran 60 perfusate					

Sarrafzadeh et al. (17)	Single catheter in territory where aneurysm located	Not specified	IL-6 levels in CMD and CSF were higher than serum	N/A	Not specified	IL-6 levels are elevated in CMD and CSF post-aSAH
	*IL-6*	Unclear surgical Tx for aneurysm	IL-6 levels in CSF, CMD, and serum were higher in those with symptomatic vasospasm but was not predictive			
	2–3 times daily for 10 days	Some received DC	However, CMD and CSF IL-6 levels were higher in those presenting with acute deficits and predicted the development of further DIND secondary to vasospasm on day 7 post-bleed (*p* = 0.025)			IL-6 CMD levels may be predictive of DIND secondary to vasospasm in those presenting with acute deficits
	Ringer’s perfusate	10 developed DIND secondary to vasospasm—Tx unclear				

Schiefecker et al. (18)	Mixed locations	Not specified	Patients were categorized into low-grade or high-grade inflammation based on median CMD IL-6 levels	*CMD probe location*: peri-lesional location associated with high IL-6 levels (*p* = 0.002)	Not specified	CMD IL-6 levels are higher in peri-lesional areas and in patients with ICH post-aSAH
	*IL-6*		Brain extracellular TAU-protein levels (*p* = 0.001), metabolic distress, and delayed cerebral infarction (*p* = 0.001) were linked to high-grade neuroinflammation			
	Unclear sampling interval		*Outcome: high*-grade neuroinflammation was a predictor for worse outcome three months after ictus, independently from probe location, initial H + H grade and age (*p* = 0.01)	*ICH*: presence of ICH was associated with elevated IL-6 levels (*p* = 0.003)		CMD IL-6 levels may be associated with DIND and outcome at 3 months
	Unknown perfusate					

*Studies from the same Authors and Center – there may be duplicated patient information.

*aSAH, aneurysmal subarachnoid hemorrhage; ICH, intracerebral hemorrhage; IVH, intraventricular hemorrhage; mRS, modified Rankin scale; GOS, Glasgow outcome scale; H + H, Hunt and Hess; CMD, cerebral microdialysis; LP, lumbar puncture; ICP, intracranial pressure; CSF, cerebrospinal fluid; LPR, lactate:pyruvate ratio; DC, decompressive craniectomy; IL, interleukin; a, alpha; b, beta; g, gamma; TNF, tumor necrosis factor; INF, interferon; MIP, macrophage inflammatory proteins; TNFR, tumor necrosis factor receptor; VEGF, vascular endothelial growth factor; FGF, fibroblast growth factor; DIND, delayed ischemic neurological deficit; TBI, traumatic brain injury; ICU, intensive care unit*.

**Table 4:** CSF cytokine measures and outcomes.

Reference	Interval of cytokine measure	CSF cytokines measured	Interventional therapies applied during measurement	Outcome of interest	Other interesting CSF cytokine-related outcomes	Conclusions
**PATIENT FUNCTIONAL OUTCOME**						
Chou et al. (19)	EVD-based sampling	*IL-2, IL-4, IL-5, IL-6, EGF, fractalkine, PDGF-AA*	Not specified	*Outcome assessed via mRS at 6 months*:	N/A	CSF IL-4 may be associated with 6 month outcome
	Unclear sampling interval		Unclear clip vs. coil numbers	IL-4 (*0* = 0.02) associated with good 6-month mRS		
				No association between CSF cytokines and vasospasm		

Graetz et al. (10)	EVD-based sampling	*IL-6*	Protocolized therapies directed toward ICP/CPP and vasospasm monitoring *via* TCD	*Outcome assessed via dichotomized GOS at 3 and 6 Months (good* = *4 or 5; poor* = *1–3)*	*Vasospasm*:	CSF IL-6 may be associated with outcome at 6 months
	Q8 hours for days 0–4		Unclear clip vs. coil numbers	CSF IL-6 on days 5–9 post-bleed were associated with 6 month outcome (*p* < 0.05)	No correlation between CSF IL-6 and DIND	
	Q12 hours for days 5–10				*Micodialysis based Ischemia (LPR > 30, glutamate > 80 μmol/L)*:	
					No association between CSF IL-6 and CMD-based ischemia	

Gruber et al. (20)	EVD-based sampling	*sTNFR-I, IL-1ra, TNF-a, TNF-b, IL-1a, IL-1b*	15 patients clipped	*Outcome assessed via GOS at 6 months*:	*Vasospasm*:	CSF IL-1ra, sTNFR, IL-6 may be associated with poor outcome at 6 months
	Day 1, 3–5, 6–8, 9–11 post-bleed		Otherwise not specified	Elevated CSF IL-1ra (*p* < 0.001), sTNFR (*p* = 0.02), and IL-6 (*p* = 0.001) were associated with outcome	IL-1ra correlated to DIND (*p* = 0.04)	
					IL-1ra peaked ~day 6 post-bleed and then decreased in good grade patients, while it remained elevated in poor grade patients	

Höllig et al. (21)	EVD-based sampling	*IL-6, LIF, E-selectin, ICAM-1*	Not specified	*Outcome assessed via dichotomized mRS (good* = *0–2; poor* = *3–6)*	N/A	CSF LIF at day 1 post-admission may be associated with outcome a discharge
	At day 1 only		18 patients clipped	*Outcome at Discharge*:		
				CSF LIF was associated with discharge outcome		
				*Outcome at 6 months*:		
				None of the measured cytokines were associated with outcome		

Mathiesen et al. (22)	EVD-based sampling (control group had banked LP CSF)	*IL-1a, IL-1b, IL-1ra, TNF-a*	Not specified	*Outcome assessed via dichotomized GOS at unspecified interval (good* = *4 or 5; poor* = *1–3)*	*Vasospasm*:	CSF IL-1ra and TNF-a measured at day ~3–11 post-bleed may be associated with outcome
	Unclear sampling intervals		Unclear clip vs. coil numbers	Elevated CSF IL-1ra (*p* < 0.05) and TNF-a (*p* < 0.05) at days 3–11 were associated with poor outcome	CSF IL-1ra was elevated in all patient with DIND (*n* = 3)	
					*Controls*:	
					All CSF cytokines were elevated compared to control samples	

Nakahara et al. (23)	EVD-based sampling	*HMGB-1, IL-6, IL-8, TNF-a*	Not specified	*Outcome assessed via dichotomized GOS at 3 months (good* = *4 or 5; poor* = *1–3)*	N/A	CSF HMGB-1, IL-6, IL-8, and TNF-a may be associated with outcome at 3 months
	Day 3, 7, and 14 post-admission		All underwent clipping	CSF HMGB-1, IL-6, IL-8, and TNF-a were elevated in the poor outcome group		

Niwa et al. (24)	EVD-based sampling	*IL-6, MCP-1, IL-10, MIG*	Not specified	*Outcome assessed via dichotomized GOS at 3 months (good* = *4 or 5; poor* = *1–3)*	N/A	CSF IL-6 may be associated with outcome at 3 months
	Daily for 14 days		All underwent clipping	Peak IL-6 was associated with poor outcome		

Provencio et al. (25)	EVD-based sampling	*IL-1a, IL-1ra, IL-2, IL-8, IL-17, TNF-a, INF-g*	Not specified	*Outcome assessed via dichotomized mRS at unspecified interval (good* = *1–2; poor* = *3–5)*	*N/A*	CSF IL-1a, IL-1ra, IL-2, IL-8, IL-17, TNF-a, and INF-g may be associated with outcome at 3 months
	Daily for first 3 days		Unclear clip vs. coil numbers	Elevated CSF levels of IL-1a, IL-1ra, IL-2, IL-8, IL-17, TNF-a, and INF-g were found in the poor outcome group (all *p* < 0.05)		

Sokół et al. (26)	EVD-based sampling (control group—non-ill patients with banked LP CSF)	*HMGB-1*	Not specified	*Outcome assessed via dichotomized GOS at 3 months (good* = *4 or 5; poor* = *1–3)*	*Controls*:	CSF HMGB-1 may be associated with poor outcome
	Day 1, 5, and 10 post-bleed		All coiled	CSF HMGB-1 levels were elevated at all 3 time points in those with poor outcome. Levels above 10 ng/mL were found in all with poor outcomes	SAH patients had higher HMGB-1 levels compared to controls	

Wada et al. (27)	LD-based sampling	*G-CSF*	Not specified	*Outcome assessed via mortality at unspecified interval*	*Vasospasm*:	CSF G-CSF levels may be associated with mortality
	Day 1, 3, 6, and 9 post-admission		8 clipped	Day 1 elevated G-CSF levels were associated with mortality	No correlation between CSF G-CSF levels and vasospasm	
**SHUNT DEPENDENCY STUDIES**						
Douglas et al. (28)	EVD-based sampling	*TGF-b1, TGF-b2*	Not specified	*Hydrocephalus as measured via F/U CT at 2 months*:	*Controls comparison*:	CSF TGF levels within the acute phase post-aSAH may predict chronic communicating hydrocephalus
	Q2 day sample intervals (control samples collected from 7 patients with non-hemorrhagic communicating hydrocephalus)		Unclear clipping vs. coiling numbers	CSF total TGF levels were higher in those patients whom developed CT-based hydrocephalus (*p* < 0.05)	CSF TGF levels were higher in aSAH patients vs. controls	

Kitazawa and Tada (29)	Cisternal CSF or LD sampling	*TGF-b1*	Not specified	*Shunt dependency at 3 months*:	N/A	CSF TGF-b1 levels during the second week post-aSAH may be associated with the development of ventriculomegaly and VPS dependency
	Unclear sampling interval up to day 17		23 clipped	No relation between CSF TGF-b1 and CT based peri-ventricular Hounsfield units		
				CSF TGF-b1 on days 9–17 were higher in those whom developed ventricular dilatation on CT (*p* < 0.02) and VPS dependency (*p* < 0.02)		

Takizawa et al. (30)	LP at day 14 post-bleed	*IL-1b, IL-6, TGF-b1*	Not specified	*Shunt dependency at unspecified interval*:	*Control comparison*:	CSF TGF-b1 levels at 2 weeks post-bleed may be associated with shunt dependency
	Control samples collected *via* LP		Unclear coil vs. clip numbers	TGF-b1 levels were higher in those requiring a VPS	CSF levels of all cytokines were higher in the aSAH group	

Wostrack et al. (31)	EVD-based sampling	*IL-6*	Not specified	*Shunt dependency at unspecified interval*:	*N/A*	CSF IL-6 levels may be associated with VPS dependency
	Q2 days for 14 days		Unclear coil vs. clip numbers	CSF IL-6 > 10,000 pg/mL was associated with VPS dependency (*p* = 0.009)		
**NIL ASSOCIATION STUDIES**						
Gaetani et al. (32)	Cisternal CSF gathered at surgery	*IL-6, IL-8, MCP-1, E-selectin*	Not specified	*Vasospasm*:	N/A	CSF IL-6, IL-8, MCP-1, E-selectin are not associated with vasospasm
			All were clipped	No association between measured CSF cytokines and development of vasospasm (TCD MCA > 160 cm/s)		

Kaestner and Dimitriou (33)	EVD-based sampling	*TGF-b1, TGF-b2*	Not specified	*Chronic hydrocephalus (defined on CT and need for VPS; followed for 6 months post-bleed)*:	*N/A*	CSF TGF-b1 and TGF-b2 levels are not associated with post-aSAH hydrocephalus or VPS dependency
	Daily for 10 days		Unclear coil vs. clip numbers	No correlation between CSF TGF levels with hydrocephalus and VPS dependency		

Kim et al. (34)	EVD or LD sampling	*MIP-1*	Not specified	*Outcome assessed via dichotomized mRS (good* = *0–3; poor* = *4–6) at discharge*:	*Vasospasm*:	CSF MIP-1 does not predict discharge outcome or vasospasm
	Daily up to day 14		Unclear clip vs. coil numbers	CSF MIP-1 was not predictive of outcome	CSF MIP-1 provides unclear prediction of vasospasm post-aSAH	

Kwon and Jeon (35)	EVD-based sampling	*IL-1b, IL-6, TNF-a*	“Triple H therapy”; not otherwise specified	*Outcome assessed via dichotomized GOS at unspecified interval (good* = *4 or 5; poor* = *1–3)*	*Vasospasm*:	CSF IL-1b, IL-6, and TBF-a do not correlate with outcome at 6 months
	Unclear intervals		Unclear clip vs. coil numbers	None of the measured cytokines were associated with outcome	CSF IL-6 levels were higher in the DIND group (*p* < 0.05)	

Shoch et al. (36)	EVD-based sampling	*IL-6*	Not specified	*Vasospasm (as assessed via TCD)*:	N/A	CSF IL-6 is not associated with patient outcome
	Daily for 14 days		65% treated *via* coiling	Elevated peak CSF IL-6 on day 6 post-bleed was associated with TCD-defined vasospasm		CSF IL-6 may predict TCD vasospasm and subsequent DIND
				DIND was associated with day 7 CSF IL-6 (*p* = 0.03)		
				*Outcome as assessed by dichotomized mRS at unspecified interval (good* = *0–2; poor* = *3–6)*		
				No association between IL-6 and patient outcome		

Singh et al. (37)	EVD-based sampling	*IL-1ra, IL-1a, IL-1b, IL-6, IL-8, IL-10, MCP-1, TNF-a*	Randomized to standard therapy (*n* = 7) or IV IL-1ra (*n* = 6)	*Outcome as assessed by GOS at 6 months*:	IV IL-1ra lead to a decrease in CSF IL-6 from 6 to 24 h post-bleed, compared to placebo group	CSF cytokines are not associated with patient outcome (note: studied underpowered = acknowledged in manuscript)
	Q6 hours for 24 h post infusion of IL-1ra		Unclear clip vs. coil numbers	No association between CSF cytokine factors and outcome (i.e., Decreased CSF cytokine levels with IL-1ra were not associated with outcome)		

*aSAH, aneurysmal subarachnoid hemorrhage; mRS, modified Rankin scale; GOS, Glasgow outcome scale; CMD, cerebral microdialysis; EVD, external ventricular drain; LP, lumbar puncture; VPS, ventriculoperitoneal shunt; ICP, intracranial pressure; CT, computed tomography; CSF, cerebrospinal fluid; LPR, lactate:pyruvate ratio; DC, decompressive craniectomy; IL, interleukin; a, alpha; b, beta; g, gamma; TNF, tumor necrosis factor; INF, interferon; MCP, monocyte chemoattractant protein; MIP, macrophage inflammatory proteins; TGF, transforming growth factor; EGF, epidermal growth factor; TNFR, tumor necrosis factor receptor; GM-CSF, granulocyte macrophage colony-stimulating factor; HMGB, high-mobility group box; DIND, delayed ischemic neurological deficit; CPP, cerebral perfusion pressure; TCD, transcranial Doppler; sTNFR, soluble tumor necrosis factor receptor; PDGF, platelet-derived growth factor; MCA, middle cerebral artery*.

### Bias Assessment

As the goal of this study was to produce a systematically conducted scoping review of the available literature on CMD and CSF cytokine measures in aneurysmal SAH, formal bias assessment was not done. Our desire was to produce a comprehensive overview of the current literature on the topic of CMD/CSF cytokines in aneurysmal SAH. Formal evidence grading was not conducted (given the limited and heterogenous literature body), and thus, we deemed formal bias risk assessment unnecessary for this emerging area of literature, which clearly suffers from standard biases associated with new areas of clinical research.

### Statistical Analysis

A meta-analysis was not performed in this study due to the heterogeneity of data and study design within the articles identified.

## Results

### Search Strategy Results

#### CMD Cytokine Search

**Figure 1:**
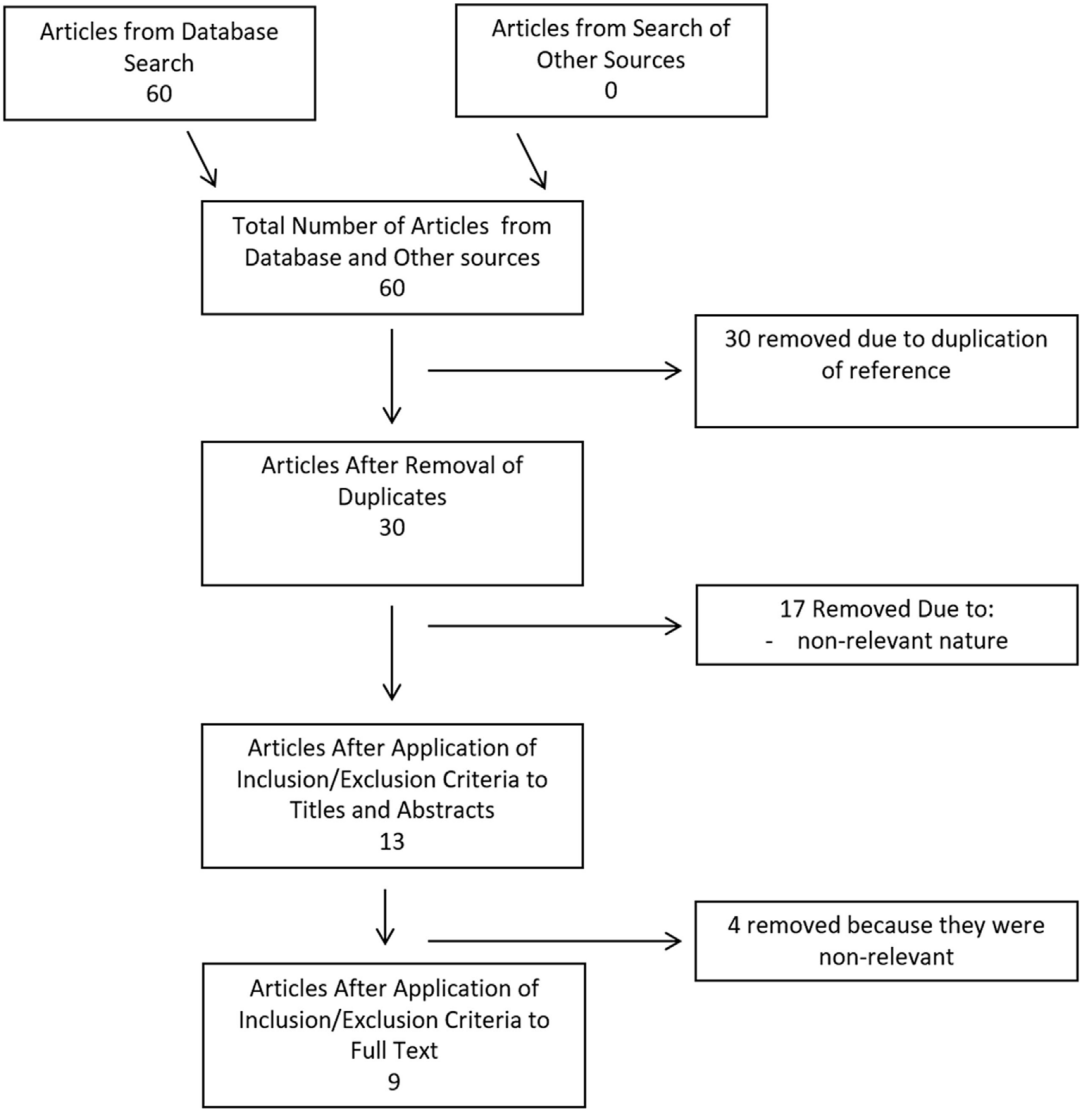
Flow diagram of search results for cerebral microdialysate cytokines.

Search strategy results for CMD cytokines in aneurysmal SAH can be seen within the flow diagram in Figure 1. At total of 60 references were returned, all coming from the database search and none identified *via* meeting proceeding searches. After duplicate removal, there were 30 articles left for the assessment *via* the first filtering of title and abstract content. Thirteen articles passed the first filter, requiring acquisition of the full manuscript to assess inclusion eligibility. Through assessment of the full articles, nine manuscripts were deemed eligible for inclusion in the final CMD systematic review. No articles were added from the reference sections of either review papers or the parent manuscripts included in the systematic review.

#### CSF Cytokine Search

**Figure 2:**
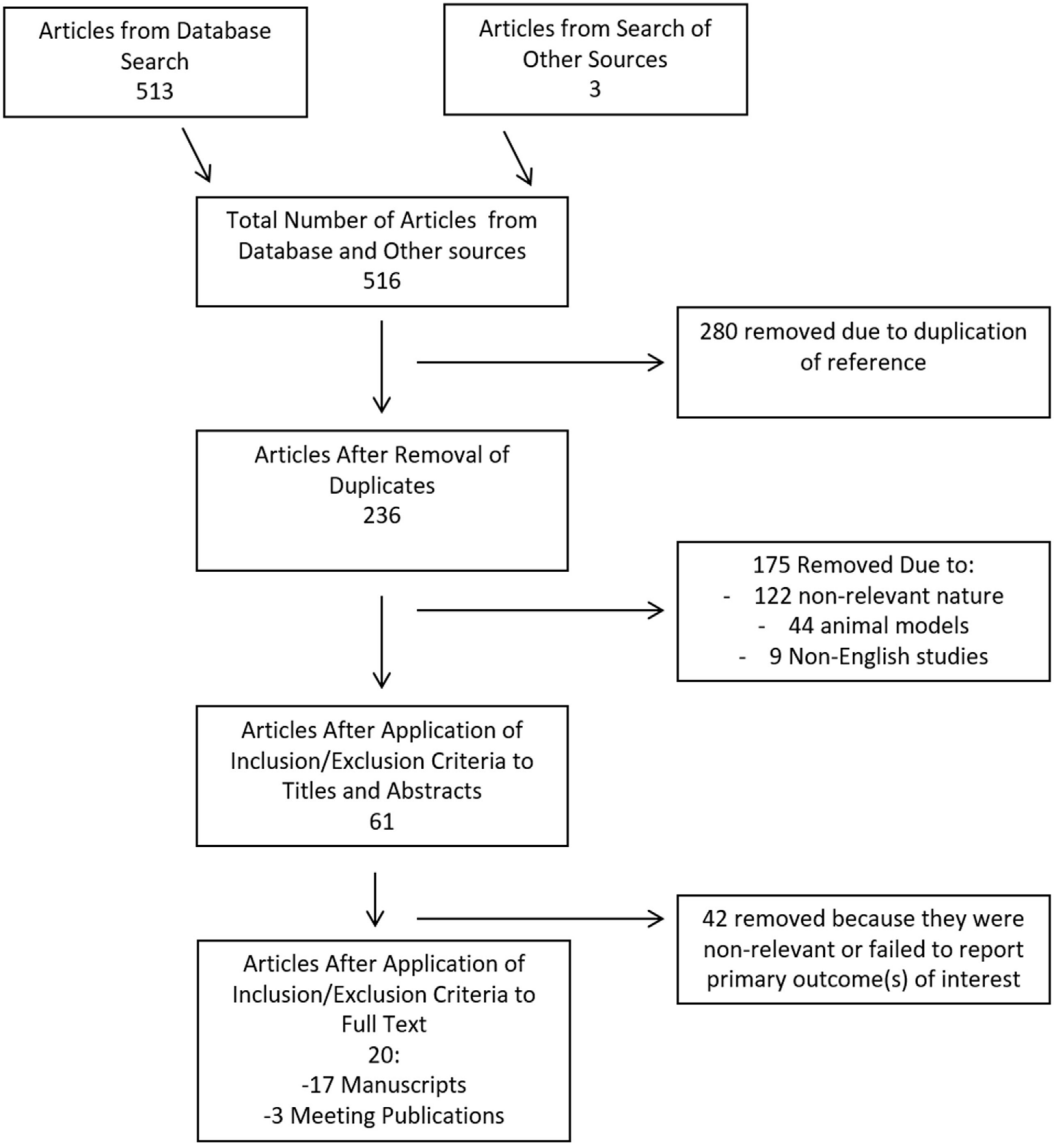
Flow diagram of search results for cerebrospinal fluid cytokines.

The search strategy flow diagram for the CSF cytokine scoping systematic review can be seen in Figure 2. Overall, 516 articles were identified, with 513 from the database search and 3 from published meeting proceedings. Two hundred and eighty duplicates removed, leaving 236 references to review in the first filter. After implementation of the first filter, 61 articles were selected for assessment of their full manuscripts. One additional reference was added from the reference sections of review papers. After the second filter of full manuscripts, 20 articles were deemed eligible for final inclusion in the CSF systematic review. Remaining articles were excluded due to non-relevance.

### Patient/Study Demographics

#### CDM Cytokine Review

All of the nine articles included in the CMD cytokine portion of the systematic review all were formal manuscript publications (10–18). There were six prospective studies, with all being prospective observational studies (10, 11, 13–15, 18). Three studies were retrospective case series (12, 16, 17).

A total of 246 unique patients with SAH were described across the 9 studies included in the CMD cytokine review. Two studies reported the same group of 88 SAH CMD patients, with a focus on analyzing different cytokine measures (15, 16). We took this into account during the calculation of the total patient numbers, to avoid counting patients twice.

The patient populations described within the CMD cytokine manuscripts were heterogeneous collections of aneurysmal SAH. The majority of studies focused on patients with poor admission clinical grades, classified as Hunt and Hess (H + H) grade 3–5, or as World Federation of Neurological Surgeons (WFNS) grade 3–5; often with high Fisher CT grade hemorrhages (i.e., 3 or 4). Aneurysm locations varied between studies, with some included both anterior and posterior circulation aneurysms. When recorded, the highest percentage of aneurysms was located within the anterior circulation with: anterior communicating artery (AComm), middle cerebral artery (MCA), and posterior communicating artery (PComm) locations predominating. Three studies failed to disclose patient clinical grade, radiographic grade, and aneurysm location information (14–16). Therapies received in the ICU were not clearly specified in the majority of the studies. Similarly, aneurysm treatment technique varied between and within the individual studies, with microsurgical clipping predominating.

We believe that some of the studies included within this portion of the review may contain duplicate patient information, as marked in Tables 1 and 3. Multiple publications from the same research groups likely were conducted on the same patient populations, yielding unique and separate manuscripts on the same group of patients. However, it is important to acknowledge that it was difficult to determine, in some circumstances, whether CMD cytokine analysis was being conducted on new patient groups or existing banked samples from previous prospective studies. With that said, our goal for the CMD cytokine scoping review was to provide an overview of all available literature in the area; hence, we have included all published papers on CMD cytokines in aneurysmal SAH within this review.

#### CSF Cytokine Review

Of the 20 articles included in the CSF cytokine systematic review (10, 19–37), 17 were formal manuscript publications (10, 20–24, 26, 28–37) and 3 were meeting abstract publications (19, 25, 27). All were prospective studies, with 19 being observational studies (10, 19–36) and 1 being a randomized control trial (37).

The populations described with in the CSF cytokine studies were quite heterogeneous, similar to the CMD cytokine papers. Most studies focused on patients with poor clinical grade (i.e., H + H 3–5; WFNS 3–5), and high Fisher CT grade (i.e., 3 or 4) upon admission. Aneurysm location varied significantly between papers with both anterior and posterior circulation aneurysms included in the studies. The majority of patients had anterior circulation aneurysms with AComm, MCA, and PComm representing the three most common locations. Therapies received within the ICU were either not specified or minimally characterized, leading to potentially significant treatment heterogeneity during CSF cytokine measurement. Finally, aneurysm treatment technique was unspecified in many studies (10, 19, 22, 25, 28, 30, 31, 33–35, 37). Those studies which disclosed aneurysm treatment employed both coiling and microsurgical clipping (20, 21, 23, 24, 26, 27, 29, 32, 36).

A total of 630 patients were described across all studies included in the CSF cytokine systematic review. The mean age for each study cohort varied significantly across studies, with all studies focusing on adult aneurysmal SAH. Details surrounding patient cohort, study design, and concurrent therapies can be found in Tables 2 and 4.

We made substantial efforts to exclude duplicate patient data across studies. However, given that many of the papers came from centers of excellence for TBI research, some of the patient data may be cross reported in multiple studies. This would reduce the total overall number of unique patients slightly. It was impossible based on the information provided within the parent studies to tease out all patients, which were reported more than once.

### Cytokine Measurement Technique

#### CMD Cytokine Review

Location of the CMD catheter varied significantly between studies and was the following: mixed healthy/peri-lesional tissue in six studies (13–16, 18), territory of the aneurysm in two studies (10, 17), and unknown tissue location in two studies (11, 13). Some studies utilized paired microdialysis catheters, one in healthy and one in peri-lesional tissue (15, 16).

Analysis interval for CMD samples was as follows: every 6 h in five studies (11, 12, 14–16), every 8–12 h in one study (17), and unspecified in three studies (10, 13, 18). The duration of sample collection varied as well, with the typical collection period of 36 h to 10 days.

Numerous different panels of cytokines were evaluated within the CMD samples, across the studies included within the review. The most commonly studied cytokines included IL-1b, IL-6, IL-10, and TNF-a. Details surrounding CMD technique and catheter locations can be seen in Table 3.

#### CSF Cytokine Review

Sampling of CSF was conducted through external ventricular drains (10, 19–26, 28, 35–37), cisternal collection intraoperatively (29, 32), or by lumbar puncture (27, 29, 30, 34). Sampling and analysis frequency varied significantly from study to study with sampling occurring from daily to every 2–3 days. Duration of sampling varied as well, up to a maximum of 14 days post-ictus (31).

Like the CMD cytokine papers, the CSF cytokine papers included in this review reported the measurement of various cytokines. The most commonly measured cytokines in CSF reported were IL-1b, IL-1ra, IL-6, IL-8, TNF, TGF, and MIP. The details of CSF sampling and specific cytokines measured can be found in Table 4.

### Outcomes

#### CMD Cytokine Review

Given that the CMD cytokine portion of this review was a scoping review, providing an overview of all the available literature on CMD cytokine measures in aneurysmal SAH, the outcomes reported by the studies are quite heterogenous. They can be seen in detail in Table 3.

Three studies reported the correlation between CMD cytokines and patient outcome (10, 13, 18). All of these three studies reported a correlation between elevated CMD IL-6 levels and poor outcome at 6 months, measured using the Glasgow Outcome Scale (GOS) (10), 3 months measured using the modified Rankin scale (mRS) (*p* = 0.01) (13), or other unspecified outcome scales (*p* < 0.01) (18).

Both the presence of intraventricular hemorrhage and intracerebral hemorrhage (ICH) post-aneurysm rupture were associated with elevated CMD IL-6 (*p* = 0.003) (18) and TNF-a (11). Similarly, peri-lesional probe CMD probe location was associated with higher IL-6 levels compared to more distant probe locations (*p* = 0.002) (18).

Radiographic cerebral vasospasm was found to be associated with elevated CMD TNF-a on day 4 and 6 post-ictus in one study (*p* < 0.01) (12). Similarly, elevated total CMD IL-6 levels were found to be associated with radiographic vasospasm in one study (17). However, there was an unclear association with the development of DIND.

Many studies provided descriptions of CMD cytokine profiles and temporal patterns. Given the various cytokines measured across the studies, it is impossible to describe all of these relationships, but highlights from these analyses are presented in Table 3. Broadly speaking, the data show temporal variations in cytokine levels, with peaks in IL-1b, IL-6, IL-8, and MIP between 6 and 12 h post-bleed (14, 15). On the other hand, IL-10 levels in CMD remained constantly elevated throughout the analysis periods recorded (14, 15).

#### CSF Cytokine Review

Within the 20 papers included in the CSF systematic review (10, 19–37), we found both manuscripts which reported positive associations between CSF cytokines with patient outcome/chronic hydrocephalus/VPS dependency (10, 19–31) and studies reporting no association (32–37) (i.e., “nil association”) between CSF cytokines and the outcomes of interest for the CSF cytokine systematic review. No studies identified reported association, “nil” or otherwise, between CSF cytokine measures and tissue outcome as assessed by follow-up neuroimaging. The subsections below describe more details around these outcomes of interest, with further information found in Table 4.

##### Positive Association Studies

Fourteen papers included within the CSF cytokine review found associations between measured cytokines with both patient outcome and/or chronic hydrocephalus/VPS dependency. Ten of these reported an association between CSF cytokines and patient outcome (10, 19–27). Four papers reported an association between CSF cytokine measures and the development of chronic hydrocephalus/VPS dependency (28–31).

###### Patient Outcome

The strongest relationships between CSF cytokine levels and clinical outcome (defined using the GOS or mRS) were seen for IL-6 (10, 20, 23, 24), IL-1ra (20, 22, 25), IL-8 (23, 25), and TNF-a/sTNFR (20, 22, 23, 25). Associations between CSF levels of high-mobility group box-1 (26), G-CSF (27), LIF (21), and IL-1a (25) and poor patient outcome were also described.

###### Chronic Hydrocephalus/VPS Dependency

Four studies discussed the correlation between CSF cytokines and the development of chronic hydrocephalus/VPS dependency (28–31). One study showed that CSF TGF-b1 and TGF-b2 levels were associated with the development of hydrocephalus (defined using CT imaging) at 2 months post-bleed (*p* < 0.05) (28). A second study also documented the correlation between elevated CSF TGF-b1 during the patients ICU stay and the development of radiographic hydrocephalus or VPS dependency at 3 months (*p* < 0.02) (29). A third study confirmed that CSF TGF-b1 levels were elevated during the acute/subacute phase in those who became shunt dependent (30). Finally, one study documented that CSF IL-6 levels during the acute/subacute phase post-bleed to be associated with VPS dependency at an unclear interval (*p* = 0.009) (31).

##### Nil Association Studies

Our review identified six studies documenting a “nil association” between CSF measured cytokines in aneurysmal SAH patients and various outcomes of interest (32–37).

Four studies reported no association between various CSF cytokines and patient outcome, as reported by in-hospital mortality or GOS at 3–6 months (34–37). The cytokines reported within these studies varied significantly, with the most common “nil associations” reported for MIP (34), IL-1b (35, 37), IL-6 (35, 37), and TNF-a (35, 37). A total of 140 patients were described within these studies, compared to the 283 patients within the studies documenting a correlation between CSF measured cytokines and patient outcome (10, 18–27).

Two studies reported no association between CSF cytokine measures and TCD-based flow velocity (32, 36), while one study failed to show an association between CSF TGF and VPS dependency (33). Further detail on the “nil association” studies can be found at the bottom of Table 4.

### Complications

Within the CMD cytokine manuscripts, all manuscripts failed to report whether complications were considered within the data collection. We suspect that complication profiles are dramatically underreported within the CMD studies.

Complication reporting within the CSF cytokine studies was essentially non-existent, with the focus of these studies being the association between CSF cytokine measures and various outcomes.

## Discussion

### CMD Cytokines in SAH

The scoping systematic review on CMD cytokines in aneurysmal SAH yielded nine studies. Despite the small number of studies and patients described within, there are a few points of interest that deserve highlighting. First, CMD-based measurement of cytokines is feasible in this patient population. Second, CMD catheter location makes a difference in the levels of cytokines measured, with peri-lesional tissue producing high levels compared to distant or healthier tissue (18). Third, peaks in CMD cytokine measures may occur within the first 6–12 h for IL-1b, IL-6, IL-8 and, MIP, while IL-10 seems to remain elevated in CMD samples through the duration of the sampling periods described (14, 15). Fourth, CMD IL-6 levels may be associated with poor outcome (10, 13, 18), up to 6 months post-injury. Finally, complications related to the use of CMD catheters are underreported, and there is a concern of selective harms reporting within the literature identified.

### CSF Cytokines in SAH

The systematic review on CSF cytokines in aneurysmal SAH, focused on the association between cytokine measures with patient outcome, chronic hydrocephalus/VSP dependency, neurophysiologic outcome, or tissue outcome. We identified some interesting trends from the 20 included studies (10, 19–37). First, a broad range of cytokines or panels of cytokines were described in these studies, but the strongest associations with poor outcome were found for elevated CSF levels of: IL-6 (10, 20, 23, 24), IL-1ra (20, 22, 25), IL-8 (23, 25), and TNF-a/sTNFR (20, 22, 23, 25). Second, acute/subacute CSF levels of TGF-b1 and TGF-b2 seemed to be associated with chronic hydrocephalus or shunt dependency at 2–3 months post-bleed (28–30). Third, we were unable to identify any studies documenting an association between CSF cytokines measures in SAH with neurophysiologic or tissue-based outcomes. Fourth, despite the “positive” associations found in the previously described papers, four manuscripts found no relationship between CSF cytokines and patient outcome (34–37). The patient numbers within these studies were smaller than that in the studies describing a positive association between CSF cytokines and patient outcome, with the “nil association” studies totaling 140 patients and the “positive association” studies totaling 286 patients. Finally, the complication reporting within the CSF cytokine studies was absent. Selective reporting bias here is a major concern.

### Limitations

Despite the interesting results of these two systematic reviews, there are significant study limitations that need to be highlighted. Limitations with each separate review can be found within the subsections to follow. Two limitations affected both reviews.

First, which was eluded to within the Methods section, is the inclusion of meeting abstracts. This could be considered controversial; however, to provide the most comprehensive scoping systematic review on this relatively “new” field of research in SAH, we thought it necessary to include these studies. Furthermore, many negative studies are presented at meeting venues, never reaching manuscript form. We wished to include any of these potential negative result abstracts to reduce publication bias seen within only positive studies. Yet, one must be cautioned in overinterpreting the results of the meeting abstracts. Given the nature of these publications, the quality of evidence is low and they are subject to significant reporting biases.

Second, within both the CMD and CSF reviews, some studies had missing data points, as seen within the tables. We made two distinct and separate attempts to contact the authors for information regarding these studies (i.e., missing demographics, etc.). The first was made in November 2016, with a second attempt in January 2017. Both were met with no response *via* electronic communication. Thus, we were unfortunately left with leaving these fields as “unknown” or “uncertain” within the tables. Although this is unfortunate, as the overall picture for each study may not be complete, this tends to be the nature of systematically conducted reviews within new and emerging areas of research.

Finally, the exact details on the cytokine measurements were not clearly delineated in most studies. With little comment on what was done to reduce interassay variability, as this could contribute to conflicting results seen within the review. Some mentioned use an enzyme-linked immunosorbent assay (ELISA) for the cytokine(s) of interest, while others mentioned multiplex “plates” for and array of cytokines, without further details. Furthermore, the timing of cytokine measurement was not mentioned or taken into consideration the reported studies. Thus, there is potential for normal circadian variation in cytokine profiles to have impacted the results reported.

#### CMD Cytokine Review

First, there were a small number of heterogenous studies found for the CMD review, with some manuscripts reporting on the same patient populations based on banked CMD samples. Furthermore, all included studies described heterogeneous cohorts of aneurysmal SAH patients with varying clinical/radiographic admission grades and aneurysm locations, making summary interpretation of results difficult. Second, the ICU and surgical therapies received by these patients during CMD sample collection/processing was quite heterogeneous. Many studies failed to specify the therapies or protocols initialized within the ICU. These treatment variations may lead to substantial changes within the CMD cytokine measures. Thus, the described associations or “nil associations” may not be accurate given this potential confounder. Third, across all the studies, there was variation in CMD catheter location. This could impact the CMD cytokine measures obtained and the described relationships. In addition, the CMD perfusate, sampling frequency, use of pooled analysis, and cytokine panel/analytic platform employed varied between studies. Given this, it is impossible for us to directly compare the absolute values of cytokines and relative recovery. Thus, our reporting of the results for CMD in SAH is limited to purely descriptive. Fourth, complications associated with CMD monitoring were seldom reported. Given the total number of patients studied, it is unlikely that there were no patients suffering from complications of invasive monitoring. Finally, given the studies and results identified for the CMD review, there is likely a large publication bias, favoring only studies with positive results.

#### CSF Cytokine Review

First, there were many quite heterogeneous studies identified in the CSF cytokine review. The included papers varied by number of patients, admission clinical/radiographic grades, aneurysm location, aneurysm treatments (clipping vs. coiling), surgical interventions, ICU-based therapies offered/provided to patients, blinding during outcome assessment, primary outcome of the studies, and duration of follow-up. These limitations suggest caution when interpreting or generalizing the results of studies that describe relationships between CSF cytokine measures and patient outcomes. Second, many cytokine associations were selectively reported, making no reference to other CSF measures and the results of statistical analysis. Therefore, there may be many more “nil associations” that were not disclosed within the body of the manuscripts. Third, complication reporting was concerning within the literature identified (as mentioned above), with underreporting is suspected. Fourth, given all the above limitations and heterogeneity issues, a meta-analysis was not performed. Finally, given the overwhelming number of “positive association” studies identified, the literature likely suffers from significant publication bias.

### CMD Technical Considerations

The complexity involved in cytokine retrieval from CMD requires some brief comments regarding some potentially more “standardized” techniques. First, standard CMD catheters employ pore sizes between 20 and 100 kDa, as the goal with these devices is to measure “common” analytes such as glucose, glutamate, glycerol, lactate, and pyruvate. Although well designed for this purpose, they are ineffective for the retrieval or larger protein biomarkers, such as cytokines, where molecular weight can easily exceed these pore size. This it is critical to know the characteristics of the biomarker of interest, thus tailoring your CMD catheter to the biomarker (44). Second, the location of placement is key. Within the TBI literature, it has been well documented that CMD catheter placement in lesional vs. peri-lesional tissue yields very different profiles of “common” analyte retrieval (45). This has also been demonstrated within CMD cytokine profiles in TBI, with lesional/peri-lesional tissue expressing much high cytokine levels compared to healthy tissue (46, 47). Thus, we recommend placement of the CMD catheter within the brain adjacent to a focal lesion or territory of interest. This way, the “at risk” brain would be monitored and not the irreversibly damaged areas. Third, the rate of perfusion should remain at 0.3μl/min. Higher perfusion rates may impair the rate of uptake of these larger proteins (44), while there is not data to support improved recovery for lower rates. Fifth, the perfusate should be colloid based. Recent investigation into the type of perfusate has demonstrated that the relative rate of recovery for cytokines is improved with colloid perfusate over crystalloid (44). The exact colloid solution to use is currently unclear. Albumin solution appears to improve the relative recovery (44); however it is expensive and labor intensive to create, thus limiting its widespread applicability. Dextran-based solutions are another potential and have been applied within some of the SAH studies quoted within this review (14, 16). However, the literature surrounding the type of dextran solution to use is limited, and we cannot make any further definitive comments at this time. Sixth, it is unclear at the current time as to the impact of frequency of CMD measurement and pooling of samples. Given we do not currently have a clear idea of the temporal profile of cytokine changes in CMD fluid, we cannot give definitive recommendations regarding the sampling frequency. Although, we would expect the rate of change in focal cytokine profile be on the order of hours or longer (such as 6 to 12 h). Finally, the cytokine analysis technique requires some comment and is applicable to both CMD and CSF analysis. Both ELISA and multiplex-based techniques have been described. All techniques are subject to inter-assay variability and thus should be conducted within established laboratory settings with trained personal, comfortable with the employed technique. The use of ELISA vs. multiplex may be site dependent or study specific. Standard analytic techniques should be employed for multicenter collaborations to improved homogeneity in measurement technique. This also applied to the entire process of CMD or CSF sampling, storage, and analysis. In addition, the normal variation in cytokine profiles should be taken into account when determining sampling frequency and pooling samples for analysis. It is particularly important to ensure that between patients, the sampling frequency and employment of sample pooling is conducted in an identical manner. Circadian variation in cytokine profiles (48) could impact the interpretation of results, and thus, standardized sampling and pooling is a necessity.

### Future Directions

Given the limited literature body and the recognized limitations in study design, there exists an opportunity for further research in the area of CMD and CSF cytokines in aneurysmal SAH, but these should seek to address the limitations seen in the studies included within the two systematic reviews. First, larger cohorts of aneurysmal SAH patients with predefined stratification of injury pattern are required. Heterogeneity in hemorrhage pattern, clinical grade, and aneurysm location make the results of the above-mentioned studies difficult to interpret, even those with positive results. Large sample sizes may allow for clinical/radiographic subgroup analysis and shed further light on the association of CMD/CSF cytokines with various subpopulations of aneurysmal SAH patients. While large studies undertaken with a uniform protocol would be ideal, we need to accept that these studies will often be conducted in relatively small populations of patients across several centers. Such a multiplicity of studies could be a substantial strength in exploring the pathophysiology and outcome associations of central nervous system cytokine levels across the spectrum of aSAH if we could undertake harmonization of the studies. Consistency across multiple centers would require rigorous harmonization of studies, which would only be possible if there were clear data provided on disease characteristics, catheter location, sample processing, and measurement techniques; and all studies used a common outcome assessment (e.g., GOSE at 3 months). With the application of common data elements between studies and centers, we may be able to more closely approach harmonization. Furthermore, banking of CSF and CMD samples from various SAH studies could prove to be a useful way of increasing the sample numbers required to analyze the milieu of CSF/CMD based cytokines. Second, homogeneous ICU/surgical treatments are necessary, preferably with protocolized therapies. Including coiled and clipped patients within the same cohort of SAH patients assuredly confounds the associations between various measured cytokines and the described outcomes. In addition, including patients with ICH evacuation and those undergoing DC due to malignant edema will also impact the resulting of cytokine measures. Third, with the application of CMD, catheter location must be considered during cytokine measures. Fourth, given the large number of cytokines involved, the use of principle component analysis of large patient populations with CMD and CSF cytokine measures may prove valuable. This has been applied within the TBI literature on CMD cytokines, with interesting preliminary results (49, 50). This could potentially identify cytokine patterns of co-expression in CMD and CSF, highlighting targets for future studies and therapeutic intervention. Fifth, accurate complication documentation is required. Sixth, one persistent problem may be the use of different analysis platforms, which results in different measured concentrations. There are no easy solutions to this problem—although control plasma levels will provide some basis for harmonization, it will be difficult to get standardize levels in CSF and particularly in CMD. Finally, multicenter prospective evaluation of cytokines within CMD and CSF is necessary to improve patient recruitment and aid with spreading the substantial cost of cytokine analysis among centers. Without collaboration, single-center small studies may unfortunately fail to add to the existing literature.

## Conclusion

The evaluation of CMD and CSF cytokines is a new area of the literature in aneurysmal SAH. The two scoping systematic reviews demonstrated the following: (1) limited literature available on CMD cytokine measurement in aneurysmal SAH with some preliminary data supporting feasibility of measurement and potential association between IL-6 and patient outcome. (2) CSF levels of several cytokines may be associated with patient outcome at 3–6 months including IL-1ra, IL-6, IL-8, and TNF-a. (3) There is a small literature supporting an association between acute/subacute CSF TGF levels and the development of chronic hydrocephalus at 2–3 months. Given the preliminary nature of these data, further large prospective multicenter studies on cytokines in CMD and CSF need to be conducted.

## Author Contributions

FZ was responsible for concept, design, systematic review searches, data acquisition/extraction, data analysis, manuscript composition, and editing. ET was responsible for systematic review searches, data acquisition/extraction, data analysis, manuscript composition, and editing. MC was responsible for data analysis, manuscript composition, and editing. PH was responsible for data analysis and manuscript composition/editing. DM and AH were responsible for concept, data analysis, and manuscript composition/editing.

## Conflict of Interest Statement

FZ has received salary support for dedicated research time, during which this project was partially completed. Such salary support came from the Cambridge Commonwealth Trust Scholarship, the Royal College of Surgeons of Canada—Harry S. Morton Travelling Fellowship in Surgery, the University of Manitoba Clinician Investigator Program, R. Samuel McLaughlin Research and Education Award, the Manitoba Medical Service Foundation, and the University of Manitoba—Faculty of Medicine Dean’s Fellowship Fund. ET has received funding support from Swedish Society of Medicine (grant no. SLS-587221). MC has financial interest in a part of licensing fee for ICM + software (Cambridge Enterprise Ltd., UK). Unpaid Co-Director of Technicam Ltd., producer of Cranial Access Device used for CMD insertion. PH and AH are the director of Technicam manufacturer of the Technicam Cranial Access Device. DM has consultancy agreements and/or research collaborations with GlaxoSmithKline Ltd.; Ornim Medical; Shire Medical Ltd.; Calico Inc.; Pfizer Ltd.; Pressura Ltd.; Glide Pharma Ltd.; and NeuroTraumaSciences LLC.
