# Supplementary material for: Cerebrospinal Fluid and Microdialysis Cytokines in Aneurysmal Subarachnoid Hemorrhage: A Scoping Systematic Review
Source: Front Neurol. 2017 Aug 8;8:379. doi: 10.3389/fneur.2017.00379 (PMC5550693; doi:10.3389/fneur.2017.00379)
Supplement: Supplementary file 1 [file Data_Sheet_1.DOC]

**Appendix A: SAH CMD Cytokine Search Strategy from MEDLINE**

1. sah.mp. [mp=ti, ab, hw, tn, ot, dm, mf, dv, kw, fs, bt, id, cc, nm, kf, px, rx, ui]

2. sah.tw.

3. subarachnoid hemorrhage.mp.

4. subarachnoid hemorrhage.tw.

5. aneurysmal subarachnoid hemorrhage.mp.

6. aneurysmal subarachnoid hemorrhage.tw.

7. spontaneous subarachnoid hemorrhage.mp.

8. spontaneous subarachnoid hemorrhage.tw.

9. cytokine.mp.

10. cytokine.tw.

11. chemokine.mp.

12. chemokine.tw.

13. beta-thromboglobulin.mp.

14. beta-thromboglobulin.tw.

15. chemokine c.mp.

16. chemokine c.tw.

17. chemokine cc.mp.

18. chemokine cc.tw.

19. chemokine cx3c.mp.

20. chemokine cx3c.tw.

21. chemokine cxc.mp.

22. chemokine cxc.tw.

23. inflammatory proteins.mp.

24. inflammatory proteins.tw.

25. factor 15.mp.

26. factor 15.tw.

27. hematopoietic cell growth factor 15.mp.

28. hematopoietic cell growth factor.mp.

29. hematopoietic growth factor.tw.

30. colony stimulating factor.mp.

31. colony stimulating factor.tw.

32. stem cell factor.mp.

33. stem cell factor.tw.

34. hepatocyte growth factor.mp.

35. hepatocyte growth factor.tw.

36. interferon.mp.

37. interferon.tw.

38. interferon gamma.mp.

39. interferon gamma.tw.

40. interferon type 1.mp.

41. interferon type 1.tw.

42. interleukin 1 receptor.mp.

43. interleukin 1 receptor.tw.

44. interleukin.mp.

45. interleukin.tw.

46. interleukin 1.mp.

47. interleukin 1.tw.

48. interleukin 2.mp.

49. interleukin 2.tw.

50. interleukin 3.mp.

51. interleukin 3.tw.

52. interleukin 4.mp.

53. interleukin 4.tw.

54. interleukin 5.mp.

55. interleukin 5.tw.

56. interleukin 6.mp.

57. interleukin 6.tw.

58. interleukin 7.mp.

59. interleukin 7.tw.

60. interleukin 8.mp.

61. interleukin 8.tw.

62. interleukin 9.mp.

63. interleukin 9.tw.

64. interleukin 10.mp.

65. interleukin 10.tw.

66. interleukin 11.mp.

67. interleukin 11.tw.

68. interleukin 12.mp.

69. interleukin 12.tw.

70. interleukin 13.mp.

71. interleukin 13.tw.

72. interleukin 15.mp.

73. interleukin 15.tw.

74. interleukin 16.mp.

75. interleukin 16.tw.

76. interleukin 17.mp.

77. interleukin 17.tw.

78. interleukin 18.mp.

79. interleukin 18.tw.

80. interleukin 23.mp.

81. interleukin 23.tw.

82. interleukin 27.mp.

83. interleukin 27.tw.

84. interleukin 33.mp.

85. interleukin 33.tw.

86. Leukemia inhibitory factor.mp.

87. leukemia inhibitory factor.tw.

88. macrophage activating factor.mp.

89. macrophage activating factor.tw.

90. macrophage migrating inhibitory factor.mp.

91. macrophage migating inhibtory factor.tw.

92. immunologic transfer factor.mp.

93. immunologic transfer factor.tw.

94. lymphotoxin alpha.mp.

95. lymphotoxin alpha.tw.

96. monokine.mp.

97. monokine.tw.

98. tumor necrosis factor alpha.mp.

99. tumor necrosis factor alpha.tw.

100. oncostatin m.mp.

101. oncostatin m.tw.

102. osteopontin.mp.

103. osteopontin.tw.

104. transforming growth factor beta.mp.

105. transforming growth factor beta.tw.

106. transforming growth factor beta 1.mp.

107. transforming growth factor beta 1.tw.

108. transforming growth factor beta 2.mp.

109. transforming growth factor beta 2.tw.

110. transforming growth factor beta 3.mp.

111. transforming growth factor beta 3.tw.

112. tumor necrosis factor.mp.

113. tumor necrosis factor.tw.

114. cerebral microdialysis.mp.

115. cerebral microdialysis.tw.

116. microdialysis.mp.

117. microdialysis.tw.

118. dialysis.mp.

119. dialysis.tw.

120. MDialysis.mp.

121. MDialysis.tw.

122. md probe.mp.

123. md probe.tw.

124. microdialysate.mp.

125. microdialysate.tw.

126. dialysate.mp.

127. dialysate.tw.

128. 1 or 2 or 3 or 4 or 5 or 6 or 7 or 8

129. 9 or 10 or 11 or 12 or 13 or 14 or 15 or 16 or 17 or 18 or 19 or 20 or 21 or 22 or 23 or 24 or 25 or 26 or 27 or 28 or 29 or 30 or 31 or 32 or 33 or 34 or 35 or 36 or 37 or 38 or 39 or 40 or 41 or 42 or 43 or 44 or 45 or 46 or 47 or 48 or 49 or 50 or 51 or 52 or 53 or 54 or 55 or 56 or 57 or 58 or 59 or 60 or 61 or 62 or 63 or 64 or 65 or 66 or 67 or 68 or 69 or 70 or 71 or 72 or 73 or 74 or 75 or 76 or 77 or 78 or 79 or 80 or 81 or 82 or 83 or 84 or 85 or 86 or 87 or 88 or 89 or 90 or 91 or 92 or 93 or 94 or 95 or 96 or 97 or 98 or 99 or 100 or 101 or 102 or 103 or 104 or 105 or 106 or 107 or 108 or 109 or 110 or 111 or 112 or 113

130. 114 or 115 or 116 or 117 or 118 or 119 or 120 or 121 or 122 or 123 or 124 or 125 or 126 or 127

131. 128 and 129 and 130
